# Notes and News

**Published:** 1890-05-17

**Authors:** 


### Notes and News.

Collected and Communicated.

Lord Hartington will preside at the annual meeting of the Children's Country Holidays Fund (10, Buckingham Street, Strand), which will be held at Devonshire House, on Monday afternoon, the 19th inst.

The secretary of the Royal Medical Benevolent College, Epsom, writing to us, says: "I am desired to inform you that the following resolution has been passed by the Council of this society: 'That the best thanks of the Council of the Royal Medical Benevolent College be given to the Editor of The Hospital for his warm advocacy of the best interests of the pensioners and foundation scholars of the college.'"

The annual report of the New Hospital for Women tells of 267 in-patients, and six deaths. Fourteen patients were helped by the Samaritan Fund. The average cost of each in-patient (exclusive of rent, rates, taxes, insurance, and repairs) has been £4 18s. 11½d.; that of each out-patient, 1s. 11d.; the average contribution towards the expenses has been 1s. 9½d. by each out-patient and 15s. 1½d. by each in-patient. The balance-sheet of the building fund shows over £21,000 received. The ceremony of laying the foundation stone last May cost £290. The address of the hospital is now 144, Euston Road, N.W.

Longue Pointe Asylum, near Quebec, was destroyed by fire last week, the death roll numbering about one hundred and fifty. The behaviour of some of the poor lunatics was most painful to witness. They seemed to consider the spread of the fire a subject of supreme glee, evincing the greatest exultation in the approach of the flames, and it was not until the walls fell in over their heads that their maniacal rejoicings were silenced. All the victims were females. During the progress of the fire three nuns made an heroic attempt to rescue a sick sister from the burning buildings. The flames, however, overtook them, and all four perished. Several firemen were injured, and many others had narrow escapes. Some of the lunatics who escaped also set fire to neighbouring buildings, but no great harm was done.

The third annual report of the Mildmay Home for Patients in Advanced Consumption, at Torquay, is a somewhat sentimental production, but luckily finishes up with a balance-sheet. Twenty-three cases were treated last year, and there are at present eight cases in the Home.

The annual meeting of subscribers to Nottingham Children's Hospital was held on April 29th. The hospital has existed 21 years, and could treat more patients if funds were forthcoming for building purposes. The report is satisfactory. New rules, similar to those of the General Hospital, have been adopted.

Two letters have appeared in the *Scots Observer* in defence of Father Damien, against whom certain accusations had been brought. Why is it we are never content to let our heroes be human? Father Damien could face death; nay, more, could face life among the lepers, from devotion to his religion. Why should we pry too closely into his little deeds of obstinacy, small speeches full of bigotry? We can be martyrs on earth, but we can only be saints in heaven.

The meeting of the National Leprosy Fund was held last week at Baron de Rothschild's. The amount at the disposal of the committee was reported to be about £6,000. It was resolved that a systematic preliminary investigation by correspondence should be immediately undertaken, with the object of ascertaining from the medical officers of the leper establishments throughout the world, and from others in positions enabling them to assist in the inquiry, the actual conditions of leprosy at the present time. It was also decided that the memorial to the late Father Damien should take the form of a granite Runic cross, with medallion portrait, to be placed over his grave at Molokai. Dr. P. S. Abrahams has been appointed technical secretary to the Society.

The Samaritan Bazaar at the Portman rooms last week was a beautiful sight, and a great success. Our tall English lasses in their gay costumes looked so handsome and healthy it was impossible to resist their appeals, and the goods sold quickly. Princess Beatrice opened the bazaar and all the medical staff were introduced to her, the name of Dr. Savage, one of the founders of the hospital, being received with special enthusiasm. The stalls were exquisitely decorated with flowers, and laden with articles rich and rare. Some of the sisters from the hospital in their grey gowns, spotted net caps and white aprons, made a pretty contrast to the very fashionable audience. Certainly this was one of the brightest and best bazaars of the season.

A very different bazaar, which we beg our readers to remember, will be held at the Royal Hospital for Incurables, at Putney, on June 10th, 11th, and 12th, from two till seven o'clock. Here will be no portraits signed by celebrities, no raffles for curiosities from India; the stalls will be laden with needlework, paintings, wood-carving, &c., all done by the inmates. But if ever you have seen the poor crippled fingers that have patiently toiled at these works, they will be more full of interest to you than the handwriting of any living actor or author. Woven into the shawls, carved into the brackets sold at Putney, will be the great truths of the Gospel of suffering. To "do good" is pleasing to all of us in a vague way, but those who desire to carry out their wish should note the date of the sale of work at Putney, and make a point of attending. The hospital freely supplies everything to the patients except clothing and the small personal necessaries so essential to their comfort, for these they depend on this sale. The articles are many of them cheap and most of them useful, so those who are not blest with very long purses will yet be welcome. The Duke and Duchess of Portland will open the bazaar—the first time the Duchess has attended a ceremony of this sort since her marriage.

The following vacancies are announced this week, the amount of the salary in each case being given after the name of the institution:—

**Resident Medical Officers.**

Royal Westminster Ophthalmic Hospital, House Surgeon.

North London Consumption Hospital, Medical Officer—£40, board, etc.

Brighton and Hove Dispensary, House Surgeon—£140, rooms, etc.

Brighton and Hove Lying-in Institution, House surgeon—£120, rooms, etc.

Victoria Park Chest Hospital, House Physician—Board, etc.

Brompton Cancer Hospital, Assistant House Surgeon and Registrar—£50, board, etc.

Kent County Asylum, Second Assistant Medical Officer—£120, board, etc.

Hants County Asylum, Third Assistant Medical Officer—£100, board, etc.

Barnwood House Hospital, Junior Assistant Medical Officer—£100, board, etc.

**Non-Resident Medical Officers.**

Glasgow Royal Hospital for Children, Extra Honorary Physician.

London County Council, Medical Officer to the Fire Brigade.

Central London Throat Hospital, Three Clinical Assistants.

Hartlepool Union, Medical Officer—£30.

Birmingham Lying-in Charity, Honorary Medical Officer.

Ennistymon Union, Medical Officer—£100.

Salford Royal Hospital, Honorary Medical Officer for the Pendleton District.

---

The Middlesex Hospital issues a very full report this year, with an historical account of the origin and progress of the institution.—The annual meeting of the Devonshire Hospital was held on May 3rd, Mr. T. H. Lowthian, J.P., in the chair. During the year 2,312 patients were admitted, and of this number 11 died.—The report of the Bristol General Hospital is chiefly notable for the exact and careful way in which every penny received is acknowledged. New and increased subscriptions are earnestly asked.

---

The annual distribution of medals and certificates to the candidates who successfully passed the examinations of the National Health Society, on domestic and personal hygiene, first aid to the injured, and home nursing, took place at Grosvenor House last week, when the Duchess of Westminster made the presentations. The chair was taken by the president of the society, the Duke of Westminster, who was supported by Sir Spencer Wells, Bart., Sir Joseph Fayrer, Mr. Ernest Hart (chairman of the society), Professor Ray Lankester, F.R.S., Dr. Scolefield, Mr. Treves, Dr. Owen Lankester, etc., etc. Eight medals and about one hundred certificates were presented to the successful candidates who had attended classes held under the auspices of the Society at East Malden, Petersfield, Balham, Henley, Mildmay Park, St. James's Square (at the residence of Lord Egerton of Tatton), etc. Among the speakers who testified to the valuable work in which the society is engaged among all classes and especially among the poor, were the Rev. Mr. Haweis, the Rev. H. G. Ridgway, Mr. Frederick Treves, F.R.C.S., and Lieut.-Col. Onslow. The two last-named spoke upon the new branch of work undertaken by the society in the department of physical education, the importance of which could not be over-estimated. It was stated that the National Health Society had determined to grant diplomas of proficiency in the art and science of physical education to such teachers of gymnastics, calisthenics, and physical exercises as have fulfilled the necessary curriculum, and have passed the required examinations. The society hopes by the institution of this diploma to encourage the development of physical education in this country; to render such training precise, effectual, and scientific; to protect the public on the one hand from incompetent teachers and to establish on the other, the position of such instructors as are fully qualified. A vote of thanks, moved by Sir Joseph Fayrer, to the Duchess for her kindness in distributing the certificates and to the Duke for presiding on that occasion brought the proceedings to a close.

All who are interested in the support of hospitals by the working classes should study the growth and development of the hospital Saturday Movement in Birmingham. The movement there is something like a Hospital Saturday, because all the money raised is sent as a free gift to the institutions, and no Central organisation strives to secure for itself patronage and control by the use of other people's money as is the case at present in London and elsewhere. The result is that the Hospital Saturday Committee have the unanimous support and confidence of the working classes in Birmingham, and they undoubtedly deserve to have it. It is remarkable, but not surprising, therefore, to find, that whereas 674 contributors gave only £5,069 in 1888, 665 contributors have given £5,948 in 1890. Further, 1,000 in 1889 subscribed collectively £6,700, but 994 contributors subscribed no less than £7,584 in 1890. A truly remarkable development. The following statement is interesting:—

| | 1887 | | 1888 | | 1889 | | 1890 | |
|---|---|---|---|---|---|---|---|---|
| | Number of Contributors. | Amount. | Number of Contributors. | Amount. | Number of Contributors. | Amount. | Number of Contributors. | Amount. |
| | | £ | | £ | | £ | | £ |
| Four o'clock | 263 | 2258 | 314 | 3062 | 323 | 3256 | 326 | 3622 |
| Five ,, | 368 | 3049 | 463 | 3964 | 451 | 4264 | 465 | 4921 |
| Six ,, | 419 | 3390 | 556 | 4505 | 569 | 4993 | 565 | 5480 |
| Seven ,, | 523 | 4026 | 674 | 5069 | 666 | 5333 | 665 | 5948 |
| Eight ,, | 646 | 4676 | 815 | 5528 | 811 | 5914 | 823 | 6713 |
| Nine ,, | 785 | 5351 | 998 | 6250 | 1000 | 6700 | 994 | 7584 |

---

The thirty-first annual report of the Buffalo General Hospital is a most exhaustive one, containing a very great amount of information on all matters connected with the hospital. The hospital has not been lucky during the past year, for the laundry and two small out-houses near were destroyed by fire, causing great inconvenience in the domestic arrangements of the hospital, owing to the destruction of a large amount of clothing needed for immediate use in the wards, as well as the extra supplies of bedding, towels, &c., stored in the laundry cupboard. However, beyond the inconvenience caused, the pecuniary loss has not been very great, as the sewing societies connected with the different churches called extra meetings, and assisted by furnishing large bundles of sewing, and a number of ladies met at the hospital to sew at different times. In this way the pressing necessities were quickly relieved, while, with the exception of 200 dollars, the money to purchase the necessary material for this purpose was generously subscribed by a number of persons interested in the hospital. This fire was perhaps not wholly a disaster, for the laundry was speedily repaired, with a number of improvements, and the other out-houses rebuilt, and, as the president remarks in his report, "Out of the fire came the solution of the problem of the morgue. The generosity of our friends has enabled us to erect a brick building for ambulance, horse, and driver; in which is a post-mortem room, a morgue, and a chapel." During the year, 1,208 patients have been admitted, of whom between nine and ten per cent. were natives of Great Britain, of this number Ireland claiming quite two-thirds. An interesting table is that giving the cost of each day of treatment given a patient, from which we find that in 1889 each patient cost 1·12 dollars per day, or 7·84 dollars a-week, as compared with 9·37 dollars in 1888. It appears that the price charged for ward patients has been five dollars a-week, but the managing body have thought it necessary to raise the price of board to seven dollars—still, as they point out, below the actual cost.

